# Gene regulatory network reconstruction: harnessing the power of single-cell multi-omic data

**DOI:** 10.1038/s41540-023-00312-6

**Published:** 2023-10-19

**Authors:** Daniel Kim, Andy Tran, Hani Jieun Kim, Yingxin Lin, Jean Yee Hwa Yang, Pengyi Yang

**Affiliations:** 1 School of Mathematics and Statistics, University of Sydney, Camperdown, NSW, Australia; 2 Computational Systems Biology Unit, Children’s Medical Research Institute, University of Sydney, Camperdown, NSW, Australia; 3 Sydney Precision Data Science Centre, University of Sydney, Camperdown, NSW, Australia; 4 Charles Perkins Centre, University of Sydney, Camperdown, NSW, Australia

**Keywords:** Computational biology and bioinformatics, Systems biology

## Abstract

Inferring gene regulatory networks (GRNs) is a fundamental challenge in biology that aims to unravel the complex relationships between genes and their regulators. Deciphering these networks plays a critical role in understanding the underlying regulatory crosstalk that drives many cellular processes and diseases. Recent advances in sequencing technology have led to the development of state-of-the-art GRN inference methods that exploit matched single-cell multi-omic data. By employing diverse mathematical and statistical methodologies, these methods aim to reconstruct more comprehensive and precise gene regulatory networks. In this review, we give a brief overview on the statistical and methodological foundations commonly used in GRN inference methods. We then compare and contrast the latest state-of-the-art GRN inference methods for single-cell matched multi-omics data, and discuss their assumptions, limitations and opportunities. Finally, we discuss the challenges and future directions that hold promise for further advancements in this rapidly developing field.

## Introduction

The transcriptional regulation of genes underpins all essential cellular processes and is orchestrated by the intricate interplay of many molecular regulators^1^. At the forefront of gene regulation are transcription factors (TFs), which interact with specific regions of DNA called cis-regulatory elements (CREs), such as promoters and enhancers^2,3^. Together, the interactions between TFs, CREs, and genes form gene regulatory networks (GRNs), which govern cell identity and cell fate decisions^4^ and play an important role in the development and progression of various diseases^5^. With the advancement of high-throughput omics technologies, it has become possible to profile the many molecular features involved in gene regulation. However, the reconstruction of these networks possess significant challenges that necessitate the development of powerful and efficient computational tools to unravel the regulatory interactions of GRNs.

**Fig. 1:**
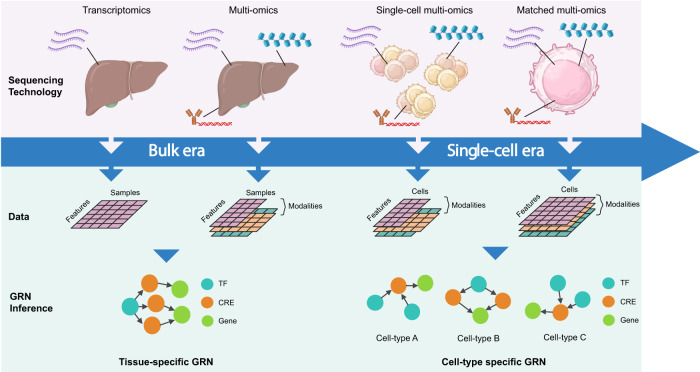
Schematic illustration of the parallel development and evolution of GRN inference and sequencing technologies. Initially, bulk sequencing technologies provided insights into regulatory interactions at the tissue level but were limited in capturing cellular heterogeneity. The emergence of single-cell technologies revolutionized the field, enabling the inference and reconstruction of cell type-specific gene regulatory networks. The advancements in sequencing technologies now allows for the multi-omic profiling of cells, offering a remarkable opportunity to precisely capture and integrate diverse molecular signals within the same cell, as shown in the cell furthest to the right of Fig. 1. Importantly, each sequencing technology possesses its own unique data structure and characteristics. For example, data of unmatched modalities do not share identical dimensions, as the cells and features, including their respective numbers, differ between each modality. Consequently, integration methods are required to map cells and features into a common space prior to GRN inference. In contrast, matched multi-modal data do not require data integration as the different modalities are captured within the same cell, which minimizes noise and thus improves the quality and accuracy of GRN inference. As a result of the developments in sequencing technologies and data structures, more accurate and comprehensive regulatory networks may be reconstructed. It is important to note that not all single-cell GRN methods reconstruct cell type or state-specific regulatory networks but instead take advantage of additional omics layers to better represent regulatory network architectures.

The earliest computational GRN inference methods were developed to leverage data from microarray and RNA-sequencing (RNA-seq) technologies, which quantitatively measure the RNA expression of whole cell populations (Fig. 1)^6^. These methods identified potential regulatory relationships by identifying co-expressed genes using measures of association, such as mutual information and correlation^7,8^. However, these methods were unable to incorporate information of the epigenetic changes that drive gene regulation, restricting their ability to assess the accessibility of regulatory binding sites, including those of TFs. These limitations were alleviated by the expansion from bulk transcriptomics to bulk multi-omics (Fig. 1) sequencing technologies such as ATAC-seq, which can be employed to identify accessible regions of chromatin that may be bound by TFs either upstream or downstream of target genes;^9^ Hi-C, a technique for measuring genome-wide chromatin conformation to capture structural changes and chromatin interactions;^10^ and ChIP-seq, which captures genome-wide protein to DNA interactions, including TF binding sites of enhancers and promoters^11^. Yet, despite their ability to uncover mechanistic insights to capture regulatory relationships more reliably, bulk sequencing technologies lack the ability to capture cell type and/or state-specific information.

The advent of single-cell omics technologies has revolutionized our ability to uncover cellular heterogeneity at the single-cell resolution (Fig. 1)^12^. Data generated by techniques such as single-cell RNA-seq (scRNA-seq)^13^, single-cell ATAC-seq (scATAC-seq)^9^, single-cell Hi-C (scHi-C)^14^, and single-cell ChIP-seq (scChIP-seq)^15^ have led to a renewed interest in developing a new generation of computational methods that can now infer regulatory relationships between regulators and their target genes at the cell type, cell state, and single-cell level^16–18^. Additionally, single-cell omics technologies have evolved from profiling single modalities (e.g., scRNA-seq, scATAC-seq) towards capturing multiple modalities at the single-cell resolution (i.e., “single-cell multi-omics”)^19^. In particular, a range of novel sequencing platforms have the ability to simultaneously profile RNA and CRE accessibility within a single cell, such as SHARE-seq and 10x Multiome^20,21^. Consequently, these technologies have led to the development of new GRN inference methods that exploit these data to further comprehensively recapitulate regulatory networks at the cell type and cell state level^22,23^.

However, navigating through the multitude of GRN inference methods and understanding how they infer regulatory connections can be a challenging task, particularly for researchers who may not have a quantitative background. Furthermore, the sheer number of available GRN inference methods can make it difficult to determine the most suitable method for a given research question of interest. To this end, we aim to assist both researchers and method developers by reviewing the methodological underpinning of GRN inference by categorizing the latest GRN inference methods developed for paired scRNA-seq and scATAC-seq data. We start by briefly describing the history of GRN inference methods and their evolution from bulk to single-cell sequencing technologies, including the underlying theoretical foundations for GRN inference that are commonly employed. For a more comprehensive overview, readers are encouraged to read previous reviews that have extensively covered earlier GRN inference methods^5,24–26^. Thus, we provide a detailed review of recent methods that reconstruct GRNs using single-cell paired multi-omic data, including their strengths and potential limitations. Finally, we discuss the current challenges of GRN inference methods and the potential directions that we hope will inspire future method development in this field.

## Methodological foundations of GRN inference

**Fig. 2:**
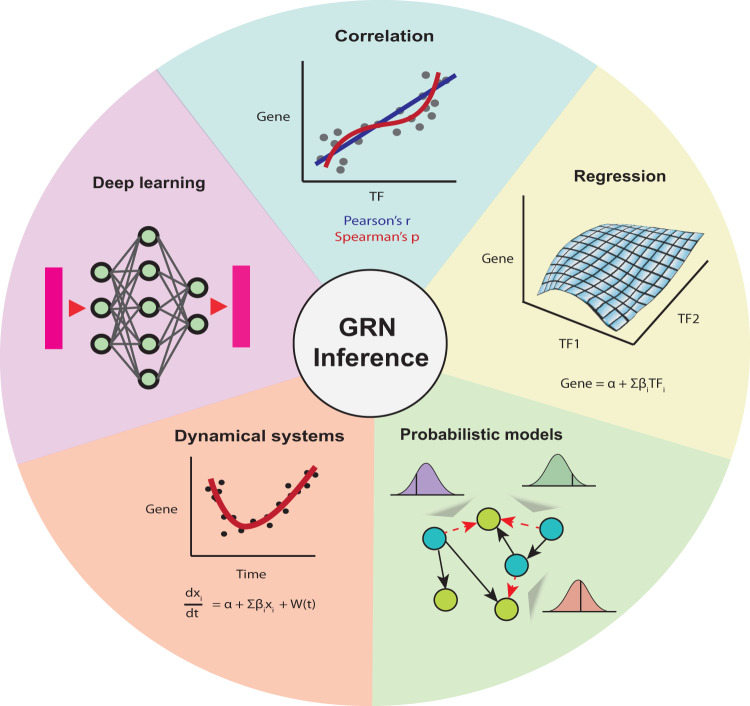
The major classes of methods for paired single-cell multi-omics GRN inference methods. Correlation-based methods seek to identify pairs of variables (i.e., TF expression, gene expression or CRE accessibility) that vary similarly. Regression-based approaches model the gene expression based on multiple predictor variables (i.e., TF expression and/or CRE accessibility). Probabilistic models aim to identify the most likely regulators for a gene. Dynamical systems-based approaches model changes in gene expression based on biological factors (e.g., TF expression, cell cycle stage, general stochasticity). Deep learning-based approaches use neural networks to infer complex relationships between TFs, CREs, genes and cells.

GRN inference relies on statistical and algorithmic principles to uncover regulatory connections between genes and their regulators. By leveraging various techniques such as correlation, regression, probabilistic models, dynamical systems and deep learning (Fig. 2), researchers can effectively model and infer regulatory architectures underlying biological systems. Here, we briefly discuss the frequently used statistical approaches and the underlying assumptions of the current GRN inference methods for paired multi-omic data.

### Correlation-based approaches

One of the most common approaches for reconstructing GRNs is motivated by the concept of “guilt by association”. In other words, genes that are co-expressed are assumed to be functionally related or co-regulated. For example, the co-expression of a TF and its putative target gene may suggest a regulatory relationship between the two. Similarly, CREs and their target genes can be determined by correlating the accessibility of CREs and expression levels of putative target genes. Commonly used measures of association include the parametric Pearson’s correlation and the non-parametric Spearman’s correlation, which can capture linear and nonlinear associations respectively (Fig. 2). Linear correlation can effectively detect relationships where an increase in TF expression or CRE accessibility leads to a proportional change in gene expression. However, nonlinear correlation can capture more complex relationships, which may better recapitulate the regulatory interactions between TFs, CREs and genes^27^. Other approaches include mutual information, a non-parametric method based on information theory, which measures the dependence between two variables^8^.

While correlation analysis may provide valuable insights into potential regulatory relationships, it is important to note that correlation alone has clear limitations. For example, correlation cannot identify which is the regulator and target if the expression levels of two TFs are correlated, nor exclude the possibility of their regulation by a third TF. Furthermore, the correlation measure will have difficulty in distinguishing direct or indirect relationships, including when confounders may be present. However, incorporating information from other modalities, such as ATAC-seq, holds the potential to alleviate these limitations as they provide additional evidence that a directional relationship between a regulator and downstream target gene exists, i.e., TF must bind to an accessible region of chromatin to regulate its target gene.

### Regression models

Regression offers an approach to capture the relationship between a response variable with multiple predictor variables. In the context of GRN inference, the response variable could be the expression of a gene, regressed on the expression or accessibility of multiple TFs and CREs, respectively (Fig. 2). By explicitly estimating the effect of each predictor onto the response (e.g., gene expression), the coefficients (e.g., TFs or CREs) from the regression model may be interpretable as strength of the association, while the sign of the coefficient can be used to infer the direction of the regulatory interactions.

In the context of inferring a GRN with ordinary least squares regression, the data can contain thousands of TFs or CREs, depending on the distance that is searched from the target gene’s transcription start site^28^. Importantly, the inclusion of a large number of predictors can often lead to overfitting, where the model becomes overly complex and generalizes poorly. Moreover, regression models can become unstable if there are correlated predictors, which is likely in a biological context given TFs can regulate each other. To address these concerns, more modern penalized regression methods such as LASSO introduces an additional penalty term based on the absolute size of the coefficients, that effectively shinks selected coefficients towards zero and thus reduces the complexity of the final estimated regulatory network. Furthermore, non-parametric approaches, such as tree-based regression, do not assume any fixed structure in the data but can be less interpretable and more computationally intensive to construct.

### Probabilistic models

Probabilistic models for GRN inference generally take the form of a graphical model, which captures the dependence between variables, such as TFs and their target genes. These approaches generally aim to model the existence and/or strength of a regulatory relationship between each TF and their putative target genes, which is estimated by finding the most probable relationships that could explain the given training data. These probabilistic measures allow for filtering and prioritization of regulatory interactions before downstream analyses, enabling more targeted investigations. However, these methods often assume that gene expression follows a specific distribution, such as a Gaussian distribution, which may not be an appropriate assumption for all genes^29^.

### Dynamical systems

While regression and probabilistic-based approaches model a response variable directly from predictor variables, dynamical systems-based approaches attempt to model the behavior of systems that evolve over time. In the case of GRN inference, one may be interested in estimating the expression of a gene with respect to various factors such as the regulatory effect of TFs, basal transcription, and general stochasticity over time (Fig. 2). These effects can be modeled as parameters in a differential equation which can be estimated from the data or literature^30^.

Dynamical-systems models carry a distinct advantage compared to previously discussed methods as they capture a diverse range of factors that can affect gene expression and its stochasticity. The estimated models are interpretable, where each parameter corresponds to a specific property. However, the complexity of larger networks and dependence on prior domain specific knowledge can make these models less scalable and prone to publication bias^31,32^.

### Deep learning models

Deep learning models are a class of machine learning techniques that have gained significant attention in recent years across a wide array of subjects, including bioinformatics^33^. These models are based on artificial neural networks which can be used in versatile architectures to perform various tasks (Fig. 2)^33–35^. For example, a multi-layer perceptron can solve regression-style problems to estimate a function, while an autoencoder can be used for dimension reduction. In particular, autoencoders can have multiple types of inputs and learn the common connections between them, representing potential regulatory relationships^36^.

However, the flexibility of deep learning approaches comes at a cost, often requiring very large training data sets as they make minimal modeling assumptions. Additionally, the constructed models can often consist of a large number of parameters, which require a substantial amount of computational resources to be estimated. Deep learning approaches are also generally considered less interpretable compared to traditional statistical models, as the fitted coefficients typically do not have a clear interpretation^37^. However, a range of recent approaches, such as saliency, aim to rectify this by identifying the important features in the overall model, which can be used to identify candidate TF regulators^38^.

## GRN inference in bulk omics era

### Bulk transcriptomics

High-throughput profiling methods such as microarray and RNA sequencing (RNA-seq) were among the first experimental methods to capture the global transcriptomic profile of a sample^39^. In response, computational methods were developed to unravel the potential regulatory connections between transcription factors and their target genes by analyzing the expression patterns of thousands of genes^40^. Notable examples include ARCANE, CLR, and MRNet, which leverage association metrics like mutual information to quantify the relationship between a TF and its target gene^41–43^. However, a key constraint of these methods lies in their pairwise calculation of association, failing to model gene expression as a function of multiple regulators. Regression-based methods, such as GENIE3, address this constraint by modeling gene expression as a function of multiple regulators, which may model regulatory relationships between regulators and target genes more accurately^44,45^. Nevertheless, an important limitation of these methods is their sole reliance on transcriptomics data, thus overlooking epigenetic modifications which are known to play a crucial role in gene regulation.

### Bulk multi-omics

The process of gene regulation and transcription has many molecular mechanisms and players, such as epigenetic modifiers, which engage in complex interactions to regulate gene expression. These molecular regulators play important roles in initiating, promoting, enhancing, and modulating gene transcription. Thus, to construct more comprehensive GRNs, it is important to include additional regulatory factors and DNA elements, such as enhancers and silencers, and structural information including chromatin conformation. For example, ATAC-seq can be used to generate more comprehensive GRNs, as used by GRaNIE, PECA, and TimeReg^46–48^. Methods such as DISTILLER and ChIP-Array 2 integrate both RNA and ChIP-seq data to identify the TFs and regulatory sequences of target genes^46,47,49–51^. Hi-C can also be used to capture the conformation of DNA and be integrated with both ATAC-seq and RNA-seq data to construct multi-omic GRNs^11,52^. Overall, the integration of various multi-omic datasets and the use of statistical models have the potential to enhance our understanding of gene regulation and uncover the dynamic interactions between TFs and their target genes in different biological contexts^30,53,54^.

Despite their advantages, both bulk transcriptomics and bulk multi-omics GRN inference methods share common limitations. Any analysis based on bulk data alone makes it challenging to infer cell type-specific information, as the omics profiles are averaged across a population of cells, thereby eliminating any signals of cellular heterogeneity^55^. However, it is well-established that various diseases, such as diabetes and cancers, are wholly or partly driven by specific cell type populations^56,57^.

## GRN inference in the single-cell era

### Single-cell omics

Many of the limitations in GRN inference from bulk omics technologies were alleviated by the birth of single-cell omics technologies. These techniques have provided a detailed glimpse into the cellular and molecular composition of diverse tissues, surpassing the capabilities of bulk sequencing methods^13,58–60^. Transcriptomics was the first to move to the single-cell level with scRNA-sequencing. Many popular GRN methods have been designed to leverage scRNA-seq data including approaches based on regression (SCENIC, scTenifoldNet), dynamical systems (SCODE) and information theory (PIDC)^22,61–63^.

Today, sequencing technologies enable the quantification of other modalities via scATAC-seq, scHi-C, and scChIP-seq, facilitating a comprehensive capture of the inter-molecular dynamics within cells^9,15,59^. Methods, such as DeepTFni, have been developed to independently leverage these additional modalities to provide an alternate approach to GRN inference^64^. Other methods aim to combine information from multiple modalities. For example, CellOracle, MICA, and IReNA use scRNA-seq and scATAC-seq separately in two stages which involves filtering putative regulatory links and then constructing the final GRN or vice versa^65–67^. Alternatively, separate GRNs can be constructed from different modalities and then combined to produce a single integrated GRN^68^.

A range of other approaches have been developed to integrate multi-omic data profiled from different cells and simultaneously learn the shared relationships between the different modalities to reconstruct regulatory networks. This includes DC3, scREG and scAI that use matrix factorization techniques to project the unmatched multi-omics data into a low-dimensional representation, thus integrating them together^69–71^. Similarly, GLUE and scTIE integrate multi-omics data by projecting the different modalities to a low-dimensional embedding, but they use an autoencoder, a deep learning-based technique that can infer complex structures from the data^72,73^. Once the low-dimensional representation that captures the shared patterns between the omics layers has been learnt, these methods use the mapping to extract multi-omic features to infer interactions (e.g., between CREs and genes), which can be used to reconstruct a GRN. These methods can also be applied to matched scRNA-seq and scATAC-seq data, by treating them as separate cell populations. However, as their main purpose is not for GRN inference, we do not review them in this article.

### Towards matched single-cell multi-omics

As the evolution from bulk RNA to bulk multi-omics involved the development and integration of additional modalities, multimodal single-cell omics technologies have led to new a wave of technologies that can profile different modalities within the same cell, often referred to as matched or paired data^74^. These technologies include SNARE-seq, which allows for the joint profiling of the transcriptome and chromatin accessibility^75^; CITE-seq, a method for capturing the transcriptome and cell surface protein markers^21^; Paired-tag, a high-throughput method for the simultaneous profiling of histone modifications and the transcriptomes^76^; and ASAP-seq, which captures the transcriptome, chromatin landscape, and protein marker expression at the single-cell resolution^77^. Importantly, these advances in sequencing technologies provide an opportunity to harness the information embedded in multimodal data that may be unattainable when integrating unmatched multi-omic data. Nevertheless, a range of computational techniques have been developed to match single cells from different modalities, or impute missing modalities, thereby increasing the availability and accessibility of multimodal single-cell data^78,79^.

**Fig. 3:**
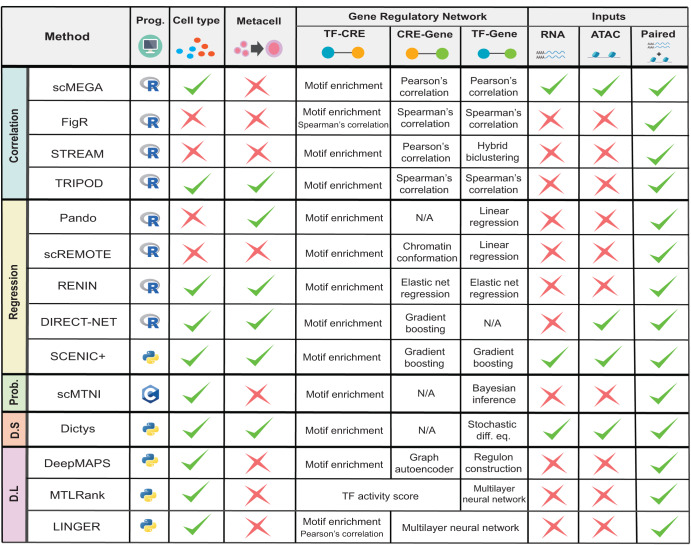
Summary of current GRN inference methods for paired multi-omic data included in this review. Prog., the program which the method was implemented in; Cell type, whether the method produces cell type-specific GRNs; Metacell, whether the method aggregates single cells into metacells (averaged expression profile of multiple similar cells); TF-CRE, the main method used to infer TF-CRE links; CRE-Gene, the main method used to infer CRE-gene links; TF-gene, the main method used to infer TF-gene links. Inputs: RNA, whether the method is compatible with scRNA-seq data alone; ATAC, whether the method is compatible with scATAC-seq data alone. All methods can take paired RNA and ATAC data as input; Prob., probabilistic model; D.S, dynamical systems; D.L, deep learning; diff. eq., differential equation. N/A cells indicate that the respective step for identifying TF-CRE, CRE-Gene, or TF-Gene links is not done for a given method. Further details of each method can be found in the section *GRN inference in the single-cell era*.

The latest GRN inference methods are designed to exploit these new data to build a more holistic model of gene regulation, thus inferring more robust and sophisticated regulatory networks. However, they vary in their approaches and complexity and not all single-cell multi-omic GRN inference methods reconstruct cell type or state -specific regulatory networks. As a result, it may be difficult to understand their differences and applicability for various contexts. Here, we categorize the latest GRN methods for paired multi-omic data into five main classes (correlation, regression, probabilistic models, dynamical systems, and deep learning) and discuss their common and distinct features. It is important to acknowledge that the categorizations do not fully encapsulate the entire statistical and methodological frameworks employed by each method, as many approaches combine multiple techniques to reconstruct GRNs. Nevertheless, by simplifying the categorizations, we intend to provide readers with a broad and accessible understanding of the underlying principles guiding these methods. A list of the methods is presented in Fig. 3. We hope that this comprehensive overview will aid researchers in navigating the current GRN inference methodological developments and facilitate informed decision-making regarding their applications.

### Correlation-based methods

**Fig. 4:**
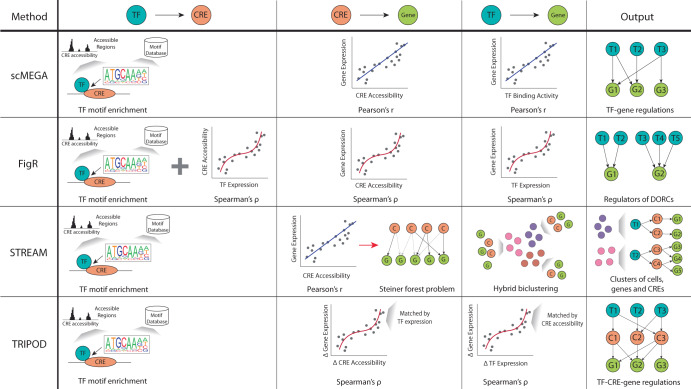
Schematic of correlation-based methods. Methods using Pearson’s correlation (scMEGA and STREAM) are restricted to detecting linear regulatory relationships while those utilizing Spearman’s correlation (STREAM and TRIPOD) can capture both linear and non-linear relationships. scMEGA, FigR and STREAM directly use the cells’ gene expression, CRE accessibility and TF expression measurements for the correlation analysis, whereas TRIPOD uses the differences between cells matched on the other component. DORCs domains of regulatory chromatin, T transcription factor, C cis-regulatory element, G gene.

These methods use correlation to infer potential regulatory relationships between pairs of regulatory elements, such as CRE vs genes or TF vs CREs (Fig. 4). Only CREs within a user-specified distance from the TSS of putative target genes are considered and inference of TF-CRE connections often include TF motif enrichment analysis (Fig. 4). While the correlation-based methods may seem similar at a glance, they have some key differences with respect to their choice of correlation metric, and implementation. For example, STREAM and scMEGA use Pearson’s correlation to capture linear relationships, whereas FigR and TRIPOD use Spearman’s correlation to capture non-linear relationships^33,59,80,81^.

FigR and STREAM aim to identify regulatory modules, which capture the key processes in a cell type or state. Briefly, FigR filters for genes with domains of regulatory chromatin (DORCs), defined as genes with a user-defined number of significantly associated CREs. Thus, FigR produces GRNs specifically composed of DORCs. Similarly, STREAM constructs networks where the modules are composed of co-expressed genes and co-accessible CREs. The most likely regulatory TF for these modules are then identified via motif enrichment analysis.

Alternatively, scMEGA and TRIPOD aim to identify individual regulatory links that make up the overall GRN. scMEGA uses TF motif enrichment and Pearson’s correlation between CRE accessibility and gene expression, including TF expression and gene expression, to select candidate TF-gene regulatory pairs. TRIPOD however, aims to find regulatory trios of TF-CRE-genes. The trios are determined by calculating the correlation of gene expression with both TF expression and CRE accessibility, while conditioning the identified CRE-gene and TF-gene associations on the other component. More precisely, CRE-gene relationships are conditioned on TF expression by matching pairs of cells with the closest TF expression values, and the differences in CRE accessibility and gene expression are used for the correlation analysis. As a result, the detected CRE-gene links will not be confounded by TF expression. Likewise, TF-gene relationships are conditioned on CRE accessibility to account for the effect that different CRE accessibilities would vary the ability for a TF to bind and thus regulate gene expression^52^.

### Regression-based methods

**Fig. 5:**
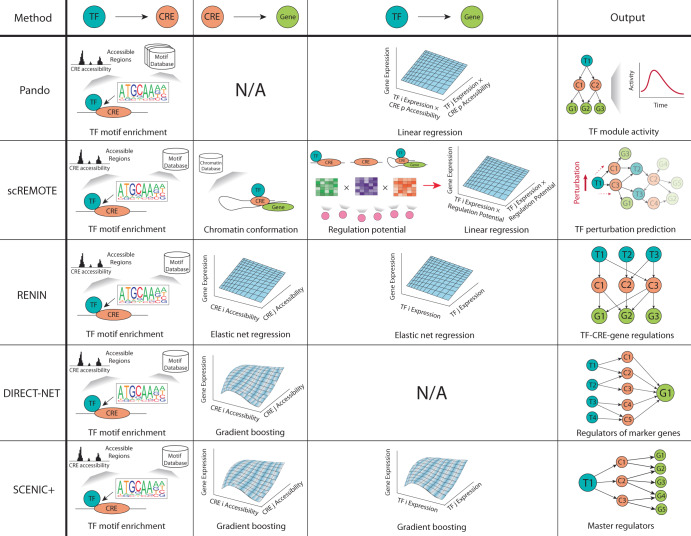
Schematic of regression-based methods. Pando, scREMOTE and RENIN are limited to the inference of linear regulatory relationships. DIRECT-NET and SCENIC+ use gradient boosting to capture non-linear relationships. T transcription factor, C cis-regulatory element, G gene.

Accounting for the fact that genes may have multiple TF regulators and vice versa, DIRECT-NET, SCENIC + , Pando, scREMOTE, and RENIN utilize regression to model gene expression as a function of multiple regulators. These methods can be further split into those that employ parametric (Pando, scREMOTE, RENIN) and non-parametric regression, such as tree-based regression (DIRECT-NET and SCENIC + ) (Fig. 5).

One approach is ordinary least squares regression, which in its simplest form assumes a linear relationship between genes and their regulators. Pando and scREMOTE model gene expression as a linear function of TF expression and CRE accessibility^23,82^. Pando estimates the regulatory effect of each TF on a gene by regressing gene expression directly on the product of CRE accessibility and TF expression while scREMOTE includes a regulation potential as a weight in the regression which is estimated from TF motif enrichment, CRE accessibility and chromatin conformation. Alternatively, RENIN uses two models with an adaptive elastic-net estimator, a regularization technique which penalizes large coefficients, resulting in a sparser regulatory network and fewer false positives^83^. The first model captures the relationship between CRE accessibility and gene expression to identify CREs that may be regulating target genes. The second models TF expression and gene expression, which incorporates the results of the first model to identify TF-gene links. In all cases, the inferred coefficients of the linear model can be interpreted as the regulatory effect of a TF on a target gene, constituting the GRN. Importantly, a clear disadvantage of Pando, scREMOTE, and RENIN is that they are limited to identifying linear relationships between regulators such as TFs and CREs and their target genes.

DIRECT-NET and SCENIC+ may mitigate this limitation as they can capture non-linear relationships by using a tree-based regression algorithm called gradient tree boosting^17,22,52^. DIRECT-NET offers a valuable functionality as it calculates the importance of each CRE’s accessibility in predicting gene expression and subsequently labels them as high, medium, or low confidence CREs before inferring TF-gene links. This allows for greater control, as only CREs of high-confidence may be kept for further downstream analyses. While both DIRECT-NET and SCENIC+ use TF motif enrichment to establish the TF-gene pairs, SCENIC+ uses an in-house generated motif compendium containing over 30,000 unique position weight matrices, where each TF has an average of 5 assigned motifs. This may have a distinct advantage in predicting TF binding sites compared to collapsing them into consensus sequences such as those used in typical motif enrichment analysis, as it may capture a broader range of TFs.

### Probabilistic models

**Fig. 6:**
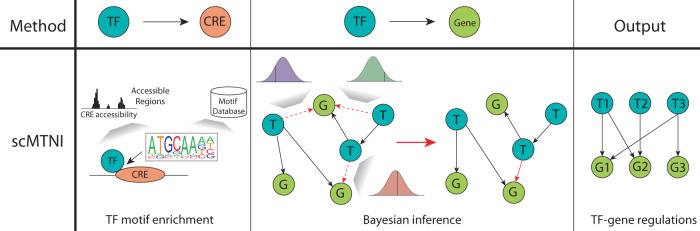
Schematic of scMTNI. scMTNI utilizes Bayesian inference to identify the most probable TF-gene regulations which constitutes the GRN. In the TF to gene step, solid arrows indicate inferred edges and dotted red arrows indicate candidate edges. The most probable candidate edge is added to the list of inferred edges. T transcription factor, C cis-regulatory element, G gene.

Unlike the methods discussed thus far, which consider target genes independently of others, probabilistic models can model the covariance between genes. To this end, Single-cell Multi-Task Network Inference (scMTNI), aims to reconstruct cell type- or condition-specific GRNs by employing a Bayesian framework and incorporating prior knowledge of the regulatory relationships when estimating cell type-specific regulatory networks (Fig. 6)^84^.

scMTNI uses a cell lineage tree to incorporate the assumption that related cell types should have similar GRNs, as well as corresponding scATAC-seq data to prioritize TF regulators that have a motif in an accessible promoter region of the target gene. The TF-gene network is inferred with a probabilistic graphical model, considering the expression of each gene as a random variable, conditioned on a set of TF regulators. The model is estimated by starting with an empty list of TF-gene regulations and iteratively adding in regulatory connections that most likely explains the expression of the target gene. Two tunable parameters allow users to constrain the number of edges in the inferred GRN and weight the importance of a TF motif in the promoter region of the gene. The final output is a GRN for each cell type in the user provided cell lineage tree. It is important to note that scMTNI assumes that gene expressions follow a Gaussian distribution, which may not be representative of biological reality^85^. Furthermore, the output of Bayesian-based approaches can be sensitive to the choice of priors, potentially limiting the robustness of the inferred GRNs^86^.

### Dynamical system-based methods

**Fig. 7:**
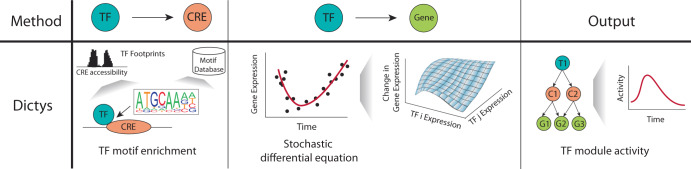
Schematic of Dictys. Dictys uses a stochastic differential equation to model gene expression as a function of multiple regulators and factors. The output can be interpreted as regulatory activity over time. T transcription factor, C cis-regulatory element, G gene.

The GRN inference methods discussed so far generally assume that the cell population of interest is sufficiently homogenous, and any variation is due to noise. However, variation among individual cells may be biologically meaningful and influenced by cell cycle and their environment. Incorporating these factors in the GRN inference process could have distinct advantages as it accounts for the dynamic nature of gene regulation and environmental interactions. In this context, Dictys is designed to capture both static and time-resolved GRNs over a trajectory using pseudo-time analysis (Fig. 7)^80^.

As opposed to the previous methods discussed so far, Dictys targets both TF footprints and motifs to establish TF-CRE links^80^, where TF footprints are smaller and thus less prone to being detected as false positives^80^. The potential regulators for each gene are then filtered to the TFs that can bind to a nearby CRE. The relationship between TFs and putative target genes are then modeled by an empirical linear model as a stochastic differential equation, where the final fitted coefficients represent the regulatory effect of the TFs on their target genes. Notably, Dictys recovers both differential regulation (logFC) and differential expression (CPM). Using differential regulation can help model changes in regulatory activity between TFs and their target genes that are not solely dependent on gene expression levels. Consequently, as Dictys models the expression over time, it may be better suited for studying differential regulatory changes within GRNs, particularly in continuous processes like cell differentiation. Additionally, Dictys may be robust to high variability due to low number of observations as it uses kernel smoothing to construct its regulatory models. However, it is important to note that like linear regression, Dictys estimates the total regulatory effect as a linear combination of individual TF expressions, which may be an oversimplification of true biological relationships, which are often more complex^81^.

### Deep learning-based methods

**Fig. 8:**
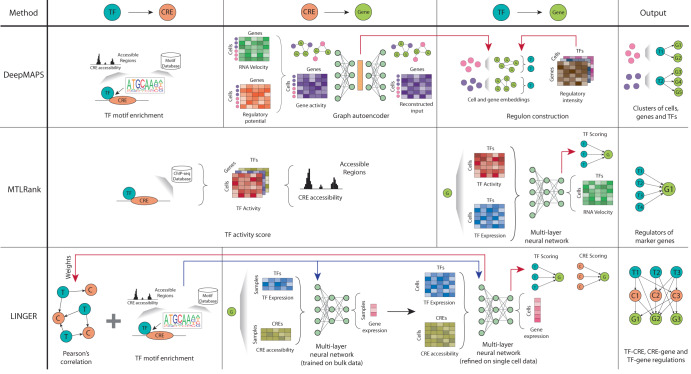
Schematic of deep learning-based methods. Both DeepMAPS and MTLRank employ the use of RNA velocity to infer regulatory relationships. While DeepMAPS identifies these networks for clusters of cells, MTLRank specifically reconstructs the regulatory connections for cell type marker genes. However, LINGER uses TF expression and CRE accessibility to predict gene expression, training on bulk data and then refining on single cell data. LINGER incorporates TF motif enrichment into the neural network structure, and uses the learnt weights to infer TF-CRE links. T transcription factor, C cis-regulatory element, G gene.

Deep learning models have gained significant attention due to their ability to learn complex non-linear patterns and shown great success in diverse domains, such as biomedical imaging, protein structure prediction, and protein function prediction^33,34^. Recent works employ deep learning models to leverage the recently available single-cell paired multi-omic data to infer regulatory networks, including DeepMAPS, MTLRank and LINGER (Fig. 8)^38,87,88^. In contrast to the other reviewed GRN inference methods, DeepMAPS and MTLRank incorporate RNA velocity, defined as the ratio of spliced and unspliced messenger RNA, which estimates the rate of change of gene expression for a given gene at the time of sequencing^89^. The regulatory impact of TFs on their target genes is rarely instantaneous and involves a cascade of regulatory events (recruitment of co-regulatory proteins and chromatin remodeling) that eventually lead to changes in gene expression. Thus, incorporating RNA velocity as a proxy for changes in gene expression over time can provide a more accurate approximation when estimating and establishing the regulatory effect of TFs on their target genes.

DeepMAPS estimates a regulatory potential for each gene in each cell by aggregating the accessibility of CREs and their proximity to the gene’s transcription start site. The regulatory potential and RNA velocity are then summarized into a gene activity matrix that captures the dynamic nature of each gene in each cell. A graph autoencoder is then used to learn a lower-dimensional embedding of both the genes and cells which is used to group clusters of cells and genes with similar gene activities. Regulatory links between genes are then established for each cluster of cells. Like most methods discussed so far, DeepMAPS uses TF motif enrichment across the CREs to infer the regulatory TFs for these clusters. In contrast, MTLRank calculates a TF activity score from ChIP-seq and scATAC-seq data to estimate the regulatory effect between each TF and gene in a cell. The TF activity is then combined with TF expression to predict the RNA velocity using a multi-layer neural network. MTLRank then ranks the TFs based on their impact on its putative target gene’s RNA velocity, which can be used to infer regulatory relationships and thus reconstruct the GRN.

Alternatively, LINGER directly uses TF expression and CRE accessibility to predict gene expression using a multi-layer neural network, incorporating TF motif enrichment. LINGER first trains the network on bulk data, which has the advantage of leveraging knowledge from atlas-scale data across many contexts. The network is then refined using the matched scRNA-seq and scATAC-seq data. Similarly to MTLRank, the regulatory importance of TFs and CREs is estimated by their impact on their putative target gene’s expression levels. Furthermore, the TF-CRE links can be inferred by the correlation between their weights in the first layer of the neural network, which enables LINGER to construct all TF-CRE, CRE-gene and TF-gene links to reconstruct the GRN.

## Challenges and opportunities

In spite of the significant advancements in GRN inference algorithms, several key limitations remain. Here, we discuss these challenges and potential opportunities for future improvements.

### Data sparsity

Single-cell data are often characterized by pronounced sparsity and noise compared to bulk data, which may impact the construction of robust GRNs^90^. For example, while the proportion of zeros in bulk data has been estimated to be around 10%-40%^91^, the proportion of zeros in single-cell data can be as high as 90%^92^. The sparsity in single-cell data can be partially attributed to technical reasons such as inefficient library preparation and sequence amplification^93^. Additionally, single-cell technologies aim to capture the expression profiles of individual cells which often exhibit low expression levels for many genes, resulting in a limited number of captured RNA transcripts. In contrast, bulk sequencing technologies aggregate the molecular expression profiles of many cells, allowing them to capture more counts but at the expense of losing heterogeneous information at the cell type level. Importantly, the presence of a high proportion of zeros in single-cell data can lead to biased and unstable estimations of gene expression correlations, further complicating the accurate inference of GRNs^94^. Many GRN inference methods aim to address these issues by aggregating multiple similar cells into metacells (averaged expression profile of multiple similar cells). However, this can lead to inflated correlations, potentially resulting in the inference of erroneous regulatory relationships^95,96^. Other strategies include imputation, where missing values are estimated using various approaches, including probabilistic models and latent space embeddings. Yet, most existing imputation approaches have been largely designed for imputation of scRNA-seq data, with limited options available for other data modalities^97^. Nevertheless, we expect significant developments in this area with the continued advancement of sequencing technologies, resulting in improved sequencing depths. Additionally, many statistical and bioinformatics methods have emerged specifically designed to handle sparse data, demonstrating the methodological advancements to manage data sparsity in GRN inference^38,98^.

### Establishing causality

Another significant challenge in GRN inference is establishing causal relationships between regulators and their target genes. A majority of methods infer regulatory relationships by some measure of association, such as correlation^99^. Similarly, regression and probabilistic approaches model the strength and direction of associations between variables^100^. Yet these metrics and models alone are insufficient to establish causal regulatory relationships due to possible confounding factors. However, integrating multiple modalities that capture different aspects of gene regulation, such as chromatin accessibility and conformation, can provide further evidence for true regulatory links. For instance, the presence of a chromatin loop between a TF binding site and its target gene suggests a regulatory relationship as it indicates that the TF can physically bind with the target gene’s regulatory regions, such as the promoter or enhancer region^54^. Additionally, experimental methods, such as perturbation or time-series experiments, offer a more direct approach for inferring regulatory links by perturbing regulators and observing changes in their respective target gene expression levels over time^101,102^. For example, it is more likely that a regulatory relationship between a TF and its target gene exists if perturbing the TF results in the repression or activation of its target gene’s expression levels. Capturing these signals within the same cells highlights the advantages of matched multi-omic data, as the relationships between the different modalities are drawn from the same biological context, enhancing the quality and accuracy of regulatory connections made.

### Validation

The validation of GRNs is a critical and open challenge given that the reconstructed GRNs aim to recapitulate biological processes of interest. Thus, GRN validation requires a thorough investigation of the concordance between the reconstructed GRNs and ‘ground truth’. To achieve this, ground truth regulatory networks inferred from wet lab experiments, such as functional perturbation experiments, are critical^103^. Loss and gain of function experiments are approaches typically used to more confidently establish regulatory connections by observing whether changes in the expression levels of a regulator results in the activation or repression of its putative target gene^101,104^. The advent of CRISPR-cas9 technologies has allowed for high-throughput screening of these regulatory interactions, significantly improving the efficiency and output of perturbation experiments^105^. Non-coding regions, such as enhancers, can also be targeted to quantify how changes in CREs might impact downstream target genes using CRISPRi enhancer tiled screens, thereby providing a means to establishing true regulatory links between CREs and target genes^106^. It is important to note that experimental validation can be costly and time consuming, and this is particularly true for matched profiling technologies. Nevertheless, advances in sequencing technologies, such as ISSAAC-seq, provide more affordable options for the joint profiling of single-cell modalities and pave the way for improved access to matched profiling technologies^107^. Thus, we expect the experimental validation of reconstructed GRNs to become more commonplace as the cost of sequencing decreases as a result of improved efficiency and sensitivity.

### Benchmarking

In the same vein, there is a need to validate and benchmark GRN inference methods to improve current limitations. GRN inference methods show considerable diversity in their reconstructed regulatory networks which is particularly evident in methods designed for single-cell data. For example, benchmarking studies of single-cell GRN inference methods have highlighted their poor accuracy and consensus on both experimental and in silico (simulated) data, particularly when increasing the number of genes considered in the inference process^24,108,109^. Not surprisingly, some methods perform better when applied to in silico compared to experimental datasets, which may be explained by the fact that in silico networks have simpler network architectures compared to true biological GRNs^110^. However, given the lack of gold standard experiments for establishing the ground truth, the use of in silico GRNs is a good intermediary option and currently a popular strategy for validating and benchmarking GRN inference methods.

The efficacy of in silico GRNs as surrogates for ground truth models is dependent on their ability to accurately model the complex direct and indirect relationships between TFs, CREs, and genes^23^. This remains a significant challenge as the underlying assumptions used to generate in silico GRNs are often oversimplifications of the underlying regulatory connections in true biological networks^110^. In silico multi-omic GRNs are also lacking, with the exception of some recent work by Li and colleagues who proposed a multi-omic GRN simulation method (scMultiSim), which aims to capture regulatory interactions between different omics layers (RNA and ATAC). While this is a significant step towards constructing more biologically accurate in silico GRNs, there are some important limitations, including the lack of output for accessible regions of chromatin. As such, there are no links between genes and regulatory domains that can act as ground truths when benchmarking multi-omic GRN inference methods. Additionally, given the absence of accessible regulatory regions and their respective sequences, it is not possible to perform TF motif enrichment analysis to infer and validate TF-CRE interactions in a reconstructed GRN.

From another perspective, evaluating reconstructed GRNs and benchmarking GRN inference methods are closely intertwined. A reliable model is one that effectively captures the characteristics of the observed data and should thus be able to produce simulated data that closely approximates the ground truth. Thus, in the context of GRN inference, an effective model should be able to generate data that accurately models the regulatory relationships between TFs, CREs, and genes. Put simply, generating a robust in silico GRN hinges on the capacity of GRN inference methods to faithfully model the ground truth, which can also be guided by experimentally validated knowledge. The current inability to achieve this suggests that the assumptions and approaches in GRN inference are not yet adequate for capturing the true complexity of GRNs. While all models inherently entail limitations and assumptions, we recommend researchers consider whether the assumptions driving the inference process of their methods are necessary and make biologically sense. This will not only improve the generalizability and accuracy of future GRN inference methods but enhance our capacity to accurately simulate the structure of single-cell multi-omic data.

## Conclusion

The parallel development of single-cell multi-omic technologies and GRN inference methods has resulted in a unique opportunity to comprehensively characterize cell type and cell-state gene regulatory relationships. As the complexity of available data increases, more powerful GRN inference methods have been developed to harness this data. In this review, we have categorized and summarized the latest state-of-the-art GRN inference methods. Correlation-based methods capture linear (scMEGA, STREAM) or nonlinear (FigR, TRIPOD) pairwise regulatory relationships. Similarly, regression-based methods identify the key TFs that explain the expression of a target gene, using linear (Pando, scREMOTE, RENIN) or nonlinear (DIRECT-NET, SCENIC + ) models. Probabilistic models (scMTNI) can incorporate prior information to identify the most likely regulators for each gene. Dynamical systems-based approaches (Dictys) incorporate external factors to model changes in gene expression over time. Finally, deep learning methods use artificial neural networks to discover complex regulatory relationships between different omics layers (DeepMAPS, MTLRank, LINGER).

GRN inference is a dynamic and rapidly evolving research field, as evidenced by the recent surge of new single-cell multi-omic GRN inference methods. Both technological advancements and algorithmic innovations will continue to drive the development of more powerful tools, leading to the discovery of novel regulatory interactions which play a crucial role in understanding the regulatory networks driving cellular identity and disease. However, while the current GRN inference methods are more advanced than previous methods, there is still work that must be done to mitigate the current limitations and improve the robustness and accuracy of inferred GRNs. Nevertheless, it is clear that both single-cell sequencing technologies and GRN inference methods have made great advances and will continue to develop to further accurately reconstruct multi-modal regulatory relationships, which will have implications for broad research areas, including health and disease.
